# The role of maternal infection in preterm birth: evidence from the Brazilian Multicentre Study on Preterm Birth (EMIP)

**DOI:** 10.6061/clinics/2020/e1508

**Published:** 2020-03-16

**Authors:** Ricardo P. Tedesco, Rafael B. Galvão, Jose Paulo Guida, Renato Passini-Júnior, Giuliane J. Lajos, Marcelo L. Nomura, Patricia M. Rehder, Tabata Z. Dias, Renato T. Souza, Jose G. Cecatti, Group Brazilian Multicentre Study on Preterm Birth Study

**Affiliations:** I Departamento de Tocoginecologia, Faculdade de Ciencias Medicas, Universidade Estadual de Campinas, Campinas, SP, BR; II Departamento de Tocoginecologia, Faculdade de Medicina de Jundiai, Jundiai, SP, BR

**Keywords:** Preterm Birth, Prematurity, Maternal Infection, Neonatal Health

## Abstract

**OBJECTIVES:**

Evidence suggests that infection or inflammation is a major contributor to early spontaneous preterm birth (sPTB). Therefore, this study aimed to investigate the development and causes of maternal infection associated with maternal and neonatal outcomes in women with sPTB.

**METHODS:**

This was a secondary analysis of a multicenter cross-sectional study with a nested case–control component, the Brazilian Multicentre Study on Preterm Birth (EMIP), conducted from April 2011 to July 2012 in 20 Brazilian referral obstetric hospitals. Women with preterm birth (PTB) and their neonates were enrolled. In this analysis, 2,682 women undergoing spontaneous preterm labor and premature pre-labor rupture of membranes were included. Two groups were identified based on self-reports or prenatal or hospital records: women with at least one infection factor and women without any maternal infection (vulvovaginitis, urinary tract infection, or dental infection). A bivariate analysis was performed to identify potential individual risk factors for PTB. The odds ratios (ORs) with their respective 95% confidence intervals were calculated.

**RESULTS:**

The majority of women with sPTB fulfilled at least one criterion for the identification of maternal infection (65.9%), and more than half reported having urinary tract infection during pregnancy. Approximately 9.6% of women with PTB and maternal infection were classified as having periodontal infection only. Apart from the presence of a partner, which was more common among women with infectious diseases (*p*=0.026; OR, 1.28 [1.03-1.59]), other variables did not show any significant difference between groups.

**CONCLUSION:**

Maternal infection was highly prevalent in all cases of sPTBs, although it was not clearly associated with the type of PTB, gestational age, or any adverse neonatal outcomes.

## INTRODUCTION

Preterm birth (PTB), defined as birth occurring before the completion of 37 weeks of gestation, is currently a priority in obstetrics and perinatal care owing to its association with neonatal death and long-term morbidity (1). The estimated global rate of PTB is 10.6% of all pregnancies. In Latin America, PTB leads to complications in 9.8% of all pregnancies. Brazil has the ninth highest PTB rate (11.2%) (2). PTB is also the leading cause of perinatal mortality. Surviving children are more likely to develop sequelae in the medium and long terms, including cerebral palsy, developmental delay, and retinopathy of prematurity, at a rate of 50–75% (1). PTB has a great impact on pregnant women and their relatives, increasing their anxiety and emotional stress levels (3).

One of the main questions is whether preterm labor and PTB can be predicted in low-risk pregnancies. PTB is a multifactorial syndrome. Potential risk factors such as low socioeconomic status, smoking, periodontitis, previous PTB, and multiple pregnancies have been previously identified. Additional factors are yet to be identified, but could be useful for the identification of women at increased risk for PTB (4). To identify the possible causes of PTB, it is necessary to differentiate between elective or provider-initiated PTB, resulting from interventions to interrupt pregnancy due to a maternal or fetal condition, and PTB after premature rupture of membranes (PROM) or due to spontaneous preterm labor, which may account for up to 50% of PTBs (5).

Some factors seem to be related to a high-risk phenotype for spontaneous PTB (sPTB), including extrauterine infection, maternal chronic disease, mid/late pregnancy bleeding, multiple pregnancy, clinical chorioamnionitis, and antepartum stillbirth. However, the majority of sPTBs occur in women with no previously identified risk factors (6).

Infection is present in up to 25% of preterm labor and 79% of extreme PTBs (5). Infectious and inflammatory processes are related to worse prognosis in preterm labor, increasing failure of tocolytic therapy, leading to higher delivery rates within 48h. PTB may actually be the only manifestation of microbial invasion of the amniotic cavity (6). Nevertheless, infections at other remote sites, e.g., urinary or periodontal infections, are also associated with sPTB (7). Inflammatory cytokines can be detected in cord blood samples collected from preterm neonates with confirmed chorioamnionitis, probably due to an altered placental and membrane microbiome that seems to trigger the onset of preterm labor and PROM (8).

Therefore, this study aimed to evaluate the development and causes of maternal infection and its association with maternal and neonatal outcomes in women with sPTB from different Brazilian regions.

## METHODS

This is a secondary analysis of the Brazilian Multicentre Study on Preterm Birth (EMIP) conducted from April 2011 to July 2012. EMIP was a multicenter cross-sectional study with a nested case–control component. The study protocol and main results have been previously reported (9-12). Currently, this analysis focuses on the development of maternal infection, type of PTB, and factors possibly associated with maternal infection and neonatal outcomes (fetal and neonatal mortality, Apgar score, birth weight, intubation, use of surfactant, neonatal intensive care unit (NICU) admission, ventilatory support, neonatal morbidity, neonatal sepsis, cerebral hemorrhage, and necrotizing enterocolitis).

Briefly, EMIP included all PTBs from April 2011 to July 2012 in 20 referral obstetric hospitals from different regions of Brazil. A random sample of women without PTB was also included in the case–control arm of the study. Data were obtained by interviewing women who had delivered during the study period while they were still in the hospital. Further information was collected from the medical records of these patients. Therefore, the criterion used for assessing maternal infection (vulvovaginitis, urinary tract infection, or dental infection) was any self-reported infectious condition or any infection identified through prenatal or hospital clinical records. Data were entered and stored in an electronic database (OpenClinica^®^), which allowed access to the entire data on all participants. Data on maternal characteristics such as age, ethnic background, obstetric history, history of infection or active infection, current treatment, chronic disease, and partnership were collected. Information on perinatal outcomes was also obtained.

All centers were included in the study only after their respective institutional review boards had approved the research project. Information from women and their neonates was collected after these women provided written informed consent. The study protocol complied with the principles applying to human research, established by the Brazilian National Health Council Resolution.

EMIP followed 33,740 women and enrolled 5,296 women, including 4,150 preterm cases (1,491 cases of sPTB, 1,191 cases of pre-labor PROM [pPROM], and 1,468 cases of provider-initiated PTB). In the current analysis, only women with sPTB and those with pPROM were included, totaling 2,682 women. Among PTB cases, two groups were identified: women with at least one infectious factor and women who did not present any evidence or self-report of maternal infection.

The analytical approach aimed to descriptively estimate the prevalence of different types of maternal infectious diseases and their relationship with PTB and assess the possible associated factors, type of PTB, gestational age at birth, and neonatal outcomes associated with maternal infection, compared to those in neonates born to women with no evidence of infectious diseases. A bivariate analysis was performed to identify possible individual risk factors for some maternal infections in PTBs. The odds ratios (ORs) adjusted using the cluster design effect with their respective 95% confidence intervals were estimated. SPSS and Stata packages were used for the analysis. The level of significance adopted was 0.05.

## RESULTS

In EMIP, the prevalence rate of PTB was 12.3% (4,150 cases in 33,740 deliveries), and sPTB or pPROM accounted for 64.6% (2,682) of these births and were included in our secondary analysis.

**Figure 1:**
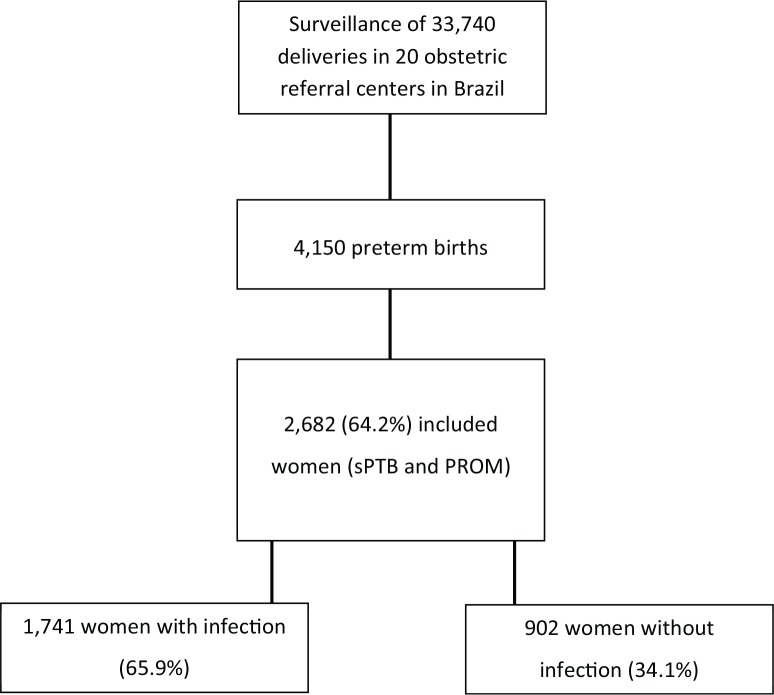
Flowchart of the enrolment of women in the study.

Among women included in the study, the majority fulfilled at least one criterion for the identification of maternal infection (65.9%) (Figure 1). In some cases, the same woman had two or more criteria for infectious disease. Table 1 shows the distribution of women with PTB who had signs of maternal infection, type of infection, and its respective prevalence. More than 50% of these women reported urinary tract infection during pregnancy and 35% had their treatment documented on medical charts.

Many women reported vaginal discharge consistent with vulvovaginitis at some stage in pregnancy (57.3%), although this diagnosis had been recorded in the medical charts of less than half of patients (26.9%). Table 1 also shows the distribution of patients according to infectious agents identified in documented cases of vulvovaginitis. Of 455 cases of periodontal infection, 287 (63%) fulfilled more than one criterion for maternal infection. Therefore, 168 women (37%) were classified as having maternal infection based on periodontal disease alone, accounting for 9.6% of the total sample of 1741 women with PTB and maternal infection.

Table 2 shows the distribution of some sociodemographic data of women with and without maternal infection. Having a partner was more frequent among women with maternal infection (77.0% *vs*. 72.3%; *p*=0.026; OR, 1.28 [1.03-1.59]). Other characteristics such as maternal age, ethnicity, family income, initial and final body mass index, and place of living did not show any significant differences between groups.

A comparison between the type of sPTB and maternal infection is shown in Table 3. Among women with maternal infection, 54.9% had sPTB, while 45.1% had pPROM. Conversely, 66.8% of women with pPROM had infection. Moreover, the distribution of the causes of PTB was similar among women with and without infection.

Gestational age at the time of PTB was similar among women with and without infection, and the majority of PTBs occurred after 34 weeks of gestation. When analyzing cases of sPTB and pPROM separately, there was no difference between both groups. Furthermore, in both groups, it was observed that most PTBs occurred after 34 weeks (Table 4).

No difference in evaluated neonatal outcomes was observed between women with and without maternal infection, as shown in Table 5. In the total of cases, there was a high prevalence rate of birth weight <2.500 g and neonatal morbidity. Additionally, 9% of neonates included in the study had a 5-min Apgar score <7, and neonatal sepsis was noted in one-third of all included neonates, and 10% stayed in the NICU for >28 days. The fetal death rate in our study was approximately 2.5% (Table 5).

## DISCUSSION

Our results did not show any significant differences in the neonatal outcomes of preterm neonates between women with or without screened infections. There was also no specific association between different types of spontaneous prematurity and presence of maternal infection. It seems that sociodemographic conditions do not influence the incidence of maternal infection, except for marital status, which was more highly associated with the development of infection in women with a partner.

It is probable that other conditions may be more important in determining neonatal outcomes, apart from a simple diagnosis of infectious disease during pregnancy. Gestational age at the time of infection, quality of neonatal care, corticosteroid administration, and other labor-related factors may be implicated in determining the overall neonatal outcome. In contrast, the prevalence rate of neonatal sepsis in our study was much higher than that reported in other studies (13). Nevertheless, it is recognized that neonatal sepsis is underreported owing to the discrepancies in its definition and surveillance worldwide (12,13). Additionally, in a recent study performed in the south of Brazil, a neonatal sepsis rate of 30.7% was responsible for neonatal mortality, which is also a high rate (14).

Furthermore, the diagnosis of maternal infection in a woman with preterm labor does not necessarily imply that it is the only cause of prematurity. It is also worth mentioning that PTB may be related to asymptomatic or occult infection (13) or even due to colonization by infectious agents without infection, e.g., group B streptococcal colonization (15). In our sample, we divided women according to the clinical development of infection, and there were no data on maternal colonization or development of asymptomatic or occult infection. The very existence of these conditions could trigger preterm labor and be responsible for the evaluated neonatal outcomes.

It is noteworthy that the criteria used for maternal infection involved only aspects that could be clinically identified during obstetric care in all participating centers. The detection of maternal infection remains of utmost importance for the prevention of preterm labor, as shown in the EMIP (10) with either bacterial vaginosis or urinary tract infection identified as risk factors for sPTB. However, it seems that maternal infection has no influence on worse neonatal outcomes. Our data support that these preterm neonates require no additional care beyond what is usually offered to preterm neonates in general.

One of the infections most commonly associated with PTB is chorioamnionitis, which is often misdiagnosed because of an asymptomatic clinical presentation (13). In this study, this condition was not specifically evaluated. As a result, information on its prevalence rate is inadequate in this population. Moreover, puerperal infection was not evaluated among study participants. We do recognize that this is one of the possible limitations of this study regarding infection, as is the case of the criteria used for identifying infection.

Our results showed a high prevalence of maternal infection among women included in the study. The high rate results from the analysis of a particular group of women with preterm labor. In part, this result was obtained because the criteria adopted for the identification of maternal infection involved self-reported data. As expected, the percentage of self-reported treatment for vaginal discharge was much higher than that in patient medical records. The prevalence of vaginal discharge in our study was much higher than those previously reported (16), and we could not assess the pathogen associated with infection. Another study conducted elsewhere reported that vulvovaginal candidiasis was the most prevalent cause of vaginal discharge (17).

It is difficult to determine which number is correct (self-referred or documented information). Nevertheless, the difference reveals the importance of careful evaluation of infectious conditions during pregnancy that may have underestimated prevalence rates in healthcare services across the country. Moreover, these differences also reveal that the quality of information in the medical records is far from being complete and totally reliable, and this represents another limitation of this current study.

In this study, maternal outcomes were not evaluated. Therefore, conclusions regarding the potential aggravation of puerperal outcomes due to maternal infection could not be drawn.

Further, this was a secondary analysis of a cohort with a sample size empowered to analyze all causes of PTBs, which failed to consider specific causes of that event, and our sample may be unpowered. Additionally, data were obtained after maternal interview, and some information may have been inaccurate. In contrast, the EMIP study was conducted in different Brazilian settings. It covered a large number of deliveries, including almost all preterm deliveries occurring in those settings during that period.

We believe that a more detailed approach is needed to strengthen the correlation between infectious processes and PTB. Furthermore, it is necessary to gain better understanding of the changes occurring in the intrauterine environment and their consequences to preterm neonates. Even if maternal infection is unrelated to PTB, we cannot dismiss the condition in clinical practice owing to its hazardous effects on maternal health. Therefore, early detection and treatment of asymptomatic or occult infection, including urinary tract infection and chorioamnionitis, is important.

## AUTHOR CONTRIBUTIONS

Passini-Júnior R, Cecatti JG and Tedesco RP initially conceived the main study and wrote the first draft of the manuscript. Passini-Júnior R, Cecatti JG, Lajos GJ, Nomura ML, Rehder PM, Dias TZ and Souza RT conducted the study and performed data collection. Tedesco RP, Galvão RB and Guida JP wrote the first draft of the manuscript. All of the authors critically reviewed the manuscript and have read the final version and agreed on the submission.

## Figures and Tables

**Table 1:** Distribution of women who had preterm birth according to the development of maternal infection, type, and treatment.

Conditions	n	%
Maternal infection		
Yes	1741	65.9
No	902	34.1
Total^a^	2643	100.0
Type of infection		
Self-reported treatment for vaginal discharge^b^	997	57.3
*Vulvovaginitis* mentioned in clinical records	469	26.9
Bacterial vaginosis	240	13.8
Candidiasis	212	12.2
Trichomoniasis	30	1.7
Others	19	1.1
Treatment for *vulvovaginitis* mentioned in clinical records	421	24.2
Self-reported treatment for UTI^c^	995	57.2
Treatment for UTI mentioned in clinical records	622	35.7
Self-reported dental infection^d^	455	26.1
Total	1741*	100.0

Missing information for ^a^39, ^b^4, ^c^13, and ^d^10 women.

* The same women may have fulfilled more than one criterion for maternal infection.

UTI, urinary tract infection.

**Table 2:** Distribution of women with preterm birth according to sociodemographic characteristics based on the presence of maternal infection.

	Maternal infection^a^			
Sociodemographic characteristics	Yes	No	*p*	OR_adj_ [95% CI]
Maternal age - mean (SD)	25.0 (6.85)	25.2 (6.95)	0.575	1.00 [0.98-1.01]
From urban area (%)^b^	89.4	91.2	0.183	0.82 [0.61-1.11]
White (%)	43.1	43.1	0.996	1.00 [0.86-1.17]
With partner (%)	77.0	72.3	**0.026**	**1.28 [1.03-1.59]**
Family income >R$ 2.000,00 (%)^c^	10.3	12.0	0.167	0.94 [0.87-1.03]
Initial BMI - mean (SD)^d^	23.55 (5.07)	23.48 (4.71)	0.713	1.00 [0.98-1.02]
Final BMI - mean (SD)^e^	27.51 (5.33)	27.29 (5.19)	0.501	1.01 [0.99-1.03]
Total^a^	1741	902		

Missing information for ^a^39, ^b^13, ^c^239, ^d^360, and ^e^457 women.

OR_adj_, odds ratio adjusted using the cluster design effect.

**Table 3:** Distribution of women according to the type of preterm birth (spontaneous preterm birth and preterm premature rupture of membranes) based on the presence of maternal infection.

	Maternal infection			
Type of PTB	Yes	No	*p*	OR_adj_ [95% CI]
Spontaneous PTB (%)	956 (54.9)	511 (56.7)	0.204	0.93 [0.83-1.04]
pPROM (%)	785 (45.1)	391 (43.3)		1.00
Total	1741	902		

OR_adj_, odds ratio adjusted using the cluster design effect.

**Table 4:** Distribution of women with preterm birth according to gestational age at delivery and type of PTB based on the presence of maternal infection.

		Maternal infection		
Type PTB	Gestational age	Yes	No	OR_adj_ [95% CI]
sPTB^p1^	<32 weeks	20.7	23.9	1.00
	32–33 weeks	13.4	15.7	0.99 [0.64-1.52]
	>34 weeks	65.9	60.5	1.26 [0.92-1.72]
pPROM^p2^	<32 weeks	17.3	19.2	1.00
	32–33 weeks	14.9	17.4	0.95 [0.67-1.35]
	>34 weeks	67.8	63.4	1.18 [0.93-1.51]
Total^p3^	<32 weeks	19.2	21.8	1.00
	32–33 weeks	14.1	16.4	0.98 [0.72-1.32]
	>34 weeks	66.7	61.8	1.23 [0.96-1.57]

*p*_1_=0.171; *p*_2_=0.198; *p*=0.078.

pPROM, pre-labor premature rupture of membranes; sPTB, spontaneous preterm birth.

OR_adj_, odds ratio adjusted using the cluster design effect.

**Table 5:** Distribution of women who had preterm birth according to neonatal results and presence of maternal infection (in percentage).

	Maternal infection		
Neonatal outcomes	Yes	No	*p*
Birth weight <2.500 g	68.0	71.6	0.135
Apgar score at 1 min <7	23.8	26.2	0.196
Apgar score at 5 min <7	9.1	9.4	0.881
Fetal death	2.1	3.2	0.098
Intubation	14.7	16.9	0.200
Use of surfactant	13.6	15.9	0.092
NICU admission >28 days	9.9	10.0	0.982
Total time admission >28 days	15.1	15.4	0.809
Ventilatory support	48.8	46.8	0.493
Neonatal morbidity	69.1	66.4	0.343
Neonatal sepsis	29.7	31.5	0.399
Cerebral hemorrhage grade zero	9.3	9.6	0.870
Necrotizing enterocolitis	1.7	3.1	0.106
Any of the above outcome	84.9	86.9	0.261

## References

[1] World Health Organization (2018). Preterm Birth. Fact Sheet Number 363, Updated February 2018.

[2] Chawanpaiboon S, Vogel JP, Moller AB, Lumbiganon P, Petzold M, Hogan D (2019). Global, regional, and national estimates of levels of preterm birth in 2014: a systematic review and modelling analysis. Lancet Glob Health.

[3] Koullali B, Oudijk MA, Nijman TA, Mol BW, Pajkrt E (2016). Risk assessment and management to prevent preterm birth. Semin Fetal Neonatal Med.

[4] Tambor V, Vajrychova M, Kacerovsky M, Link M, Domasinska P, Menon R (2015). Potential Peripartum Markers of Infectious-Inflammatory Complications in Spontaneous Preterm Birth. BioMed Res Int.

[5] Cappelletti M, Della Bella S, Ferrazzi E, Mavilio D, Divanovic S (2016). Inflammation and preterm birth. J Leukoc Biol.

[6] Combs CA, Garite TJ, Lapidus JA, Lapointe JP, Gravett M, Rael J (2015). Detection of microbial invasion of the amniotic cavity by analysis of cervicovaginal proteins in women with preterm labor and intact membranes. Am J Obstet Gynecol.

[7] Webb DA, Mathew L, Culhane JF (2014). Lessons learned from the Philadelphia Collaborative Preterm Prevention Project: the prevalence of risk factors and program participation rates among women in the intervention group. BMC Pregnancy Childbirth.

[8] Prince AL, Ma J, Kannan PS, Alvarez M, Gisslen T, Harris RA (2016). The placental membrane microbiome is altered among subjects with spontaneous preterm birth with and without chorioamnionitis. Am J Obstet Gynecol.

[9] Passini R, Tedesco RP, Marba ST, Cecatti JG, Guinsburg R, Martinez FE (2010). Brazilian multicenter study on prevalence of preterm birth and associated factors. BMC Pregnancy Childbirth.

[10] Lajos GJ, Tedesco RP, Passini R, Dias TZ, Nomura ML, Rehder PM (2015). Methodological issues on planning and running the Brazilian Multicenter Study on Preterm Birth. ScientificWorldJournal.

[11] Passini R, Cecatti JG, Lajos GJ, Tedesco RP, Nomura ML, Dias TZ (2014). Brazilian multicentre study on preterm birth (EMIP): prevalence and factors associated with spontaneous preterm birth. PLoS One.

[12] Souza RT, Cecatti JG, Passini R, Pacagnella RC, Oliveira PF, Silva CM (2019). Cluster analysis identifying clinical phenotypes of preterm birth and related maternal and neonatal outcomes from the Brazilian Multicentre Study on Preterm Birth. Int J Gynaecol Obstet.

[13] Shane AL, Sanchez PJ, Stoll BJ (2017). Neonatal sepsis. Lancet.

[14] Alves JB, Gabani FL, Ferrari RAP, Tacla MTGM, Linck Júnior A (2018). Neonatal sepsis: mortality in a municipality in Southern Brazil, 2000 to 2013. Rev Paul Pediatr.

[15] Goldenberg RL, Culhane JF, Iams JD, Romero R (2008). Epidemiology and causes of preterm birth. Lancet.

[16] Bianchi-Jassir F, Seale AC, Kohli-Lynch M, Lawn JE, Baker CJ, Bartlett L (2017). Preterm Birth Associated With Group B Streptococcus Maternal Colonization Worldwide: Systematic Review and Meta-analyses. Clin Infect Dis.

[17] Aduloju OP, Akintayo AA, Aduloju T (2019). Prevalence of bacterial vaginosis in pregnancy in a tertiary health institution, south western Nigeria. Pan Afr Med J.

